# Fraud in a population-based study of headache: prevention, detection and correction

**DOI:** 10.1186/1129-2377-15-37

**Published:** 2014-06-10

**Authors:** Bilal Ahmed, Ali Ahmad, Akbar A Herekar, Umer L Uqaili, Jahanzeb Effendi, S Zia Alvi, Arif D Herekar, Timothy J Steiner

**Affiliations:** 1 Headache Research Foundation of Pakistan, Karachi, Pakistan; 2 Department of Neuroscience, Norwegian University of Science and Technology, NO-7491 Trondheim, Norway; 3 Division of Brain Sciences, Imperial College London, London, UK

**Keywords:** Fraud, Research misconduct, Epidemiology, Headache, Pakistan, Global Campaign against Headache

## Abstract

**Background:**

In medicine, research misconduct is historically associated with laboratory or pharmaceutical research, but the vulnerability of epidemiological surveys should be recognized. As these surveys underpin health policy and allocation of limited resources, misreporting can have far-reaching implications. We report how fraud in a nationwide headache survey occurred and how it was discovered and rectified before it could cause harm.

**Methods:**

The context was a door-to-door survey to estimate the prevalence and burden of headache disorders in Pakistan. Data were collected from all four provinces of Pakistan by non-medical interviewers and collated centrally. Measures to ensure data integrity were preventative, detective and corrective. We carefully selected and trained the interviewers, set rules of conduct and gave specific warnings regarding the consequences of falsification. We employed two-fold fraud detection methods: comparative data analysis, and face-to-face re-contact with randomly selected participants. When fabrication was detected, data shown to be unreliable were replaced by repeating the survey in new samples according to the original protocol.

**Results:**

Comparative analysis of datasets from the regions revealed unfeasible prevalences and gender ratios in one (Multan). Data fabrication was suspected. During a surprise-visit to Multan, of a random sample of addresses selected for verification, all but one had been falsely reported. The data (from 840 cases) were discarded, and the survey repeated with new interviewers. The new sample of 800 cases was demographically and diagnostically consistent with other regions.

**Conclusion:**

Fraud in community-based surveys is seldom reported, but no less likely to occur than in other fields of medical research. Measures should be put in place to prevent, detect and, where necessary, correct it. In this instance, had the data from Multan been pooled with those from other regions before analysis, a damaging fraud might have escaped notice.

## Background

Research misconduct includes fabrication, falsification or plagiarism in proposing, performing or reviewing research, or in reporting research results [1]. It appears to be common: Fanelli’s 2009 systematic review and meta-analysis of survey data found almost 2% of scientific researchers admitted having fabricated, falsified or modified data or results at least once [2]. In medicine, research misconduct is historically associated with laboratory or pharmaceutical research but has been uncovered in a range of clinical and genetic studies (*e.g*, [3-11]). In such circumstances the vulnerability to misconduct of epidemiological or population-based surveys should be recognized. As such surveys are performed to assess the burden of a disease, to underpin needs assessment and inform health policy involving the allocation of usually limited resources, research misconduct and failure to detect it can have major and far-reaching implications.

With the availability of electronic data loggers, portable touch-screen computers, on-line maps and GPS trackers, data collection in many environments has become paper-free and much easier. These uses of technology have facilitated quality control over data collection, leaving fewer ways to cheat without being discovered. However, in developing countries where access to technology is limited and data collection is still mainly paper-based, multiple safeguards may need to be employed to maintain quality assurance and prevent misconduct and its consequences.

We report here how fraud in a nationwide epidemiological headache survey occurred and how it was discovered and rectified before it could cause harm. The context was a door-to-door survey to estimate the prevalence and burden of primary headache disorders in Pakistan. The protocol for the survey, designed according to standard principles [12], required data collection by hired non-medical interviewers from participants in six major cities across the four provinces of Pakistan, and from rural areas neighbouring each city. The expected procedure was to call at randomly-selected households unannounced, list the adult household members in each, select one randomly and interview that person (returning by appointment to do so if he or she was not present at the initial visit). The interview followed a structured questionnaire, including demographic enquiry, screening and diagnostic headache questions, and further enquiry into headache-attributed burden when appropriate. Full details of the survey methodology have been published previously [13]. The survey was eventually completed by 4,223 respondents.

## Methods

Measures set out within the study protocol and undertaken to ensure data integrity were preventative, detective and corrective.

### Prevention

We carefully selected and trained the interviewers, set rules of conduct for them, gave specific warnings regarding the consequences of suspected and proven falsification, provided adequate and equitable compensation, set up effective lines of communication, undertook in-field supervision during data collection, and demanded regular reporting.

At the outset of the study, we engaged an interviewer recruitment agency with experience in health-care related field surveys all over the country. We explained the purpose and design of the study. We advertised for and selected interviewers who had a track-record of reliability, could speak the local (provincial) language and could read and write in Urdu fluently, and hired them on monthly salaries. There were two interviewers in each of the six survey locations, except Lahore with four to accommodate its larger size. We called all fourteen to the main centre (Karachi) for a two-day workshop and trained them according to a set training protocol which included a) face-to-face meetings with all co-investigators and introductions to the supervising co-investigators for each location, b) the purpose and goals of the study, c) its importance and likely impact, d) an overview of headache disorders, e) administration of the structured questionnaire, f) mock interview sessions, g) a question and answer session and h) discussion and resolution of any queries. Afterwards they returned to their respective cities and the questionnaires, weighing machines, measuring tapes and stationery bags were mailed to them. All expenses were reimbursed.

One of each pair or foursome of interviewers was appointed location supervisor.

During data collection, we monitored the interviewer-groups by regular telephone calls and location supervisors provided regular updates on progress. One co-investigator was responsible for each location. We made occasional announced field visits in the more accessible locations, and used these to resolve any emerging problems, passing the experience to all other locations. Special requests to overcome cultural sensitivities (such as hiring local female health workers) were met.

The data were couriered to the principal centre in Karachi at regular intervals.

### Detection

We employed two-fold (belt-and braces) fraud detection methods at all locations: comparative data analysis, and face-to-face re-contact with randomly selected participants.

Throughout the data-collection period, completed questionnaires received in Karachi were numbered and inspected for obvious irregularities. The data were entered onto computer by the data-entry team. Comparative analyses were made between each location and the others for unexpected differences.

Re-contact consisted of one surprise-visit by the co-investigators to each location in the latter half of the data collection period. Interviewers were given short notice (no more than a few hours) of our arrival. We randomly selected 10–30 questionnaires at each location, met the interviewers and accompanied them to the respective households. At each, the interviewer waited outside, out of sight, while a co-investigator sought entry to the house, asked about the recent survey visit and requested a description of the interviewer. If the original participant was available, the interview was repeated. Second questionnaires were later compared manually with those filled by the interviewers.

We focused our attention on any location where suspicions had arisen during preventative measures or data comparison.

### Correction

Full corrective measures required that data shown to be unreliable were excluded from the survey analysis and replaced by repeating the survey in new samples according to the original protocol.

## Results

In the later stages of data collection, an interviewer at one location (Multan) reported involvement in a car accident, and requested more time because only one interviewer was working. This centre began falling behind its daily target. Since two other centres were also slightly behind target, we extended the period of data collection by two months. By the end of this extension, the Multan interviewers still had not returned their rural sample of questionnaires or those from one urban cluster-sample.

During the surprise-visit to Multan, the interviewers brought all the outstanding questionnaires but were not cooperative with the data authentication procedures. They declared themselves unavailable for the task in the near future, citing unspecified “personal reasons”. Of the random sample of addresses selected for verification, only one could be found; later it transpired that the others were falsely reported.

These circumstances inevitably created strong doubts over the authenticity of the data. Comparative data analysis revealed significant discrepancies in the Multan data: the demographics of the sample were noticeably dissimilar to those reported by the Pakistan Federal Bureau of Statistics (FBS) from the last census of Pakistan in 1999, which was extrapolated to 2006 [14] (Table 1), and the prevalences and gender-distributions of headache disorders did not match expected statistics or those from other locations. We came to the realization that the interviewers had not visited the rural areas but, instead, fraudulently filled in the questionnaires with invented data.

**Table 1:** Comparisons between fraudulent and new datasets in Multan, and national demographic statistics

		**Fraudulent data (%) n = 842**	**New data (%) n = 800**	**FBS data (%)**
**Gender**	Male	70.1	43.4	49.7
	Female	29.9	56.6	50.3
**Age (yr)**	18-29	16.9	29.5	36.4
	30-39	6.5	32.3	25.4
	40-49	64.8	20.6	20.5
	50-59	10.8	12.3	13.1
	60-65	0.7	5.4	4.6
**Marital status**	Married	90.5	82.5	n/a
	Unmarried	8.8	14.9	n/a
	Divorced	0.1	1.9	n/a
**Headache % (n)**	No headache	27.9 (235)	13.3 (106)	
	Migraine	51.4 (433)	25.4 (203)	
	male	70.7 (306)	32.0 (65)	
	female	29.3 (127)	68.0 (138)	
	TTH	20.0 (168)	46.3 (370)	
	Headache on ≥15 days/month	0.1 (1)	12.4 (99)	
	MOH	0.1 (1)	2.0 (16)	
	Undetermined	0.5 (4)	0.8 (6)	

FBS: Federal Bureau of Statistics data from 1999 survey extrapolated to 2006 [14]; TTH: tension-type headache; MOH: probable medication-overuse headache; n/a: not available.

We deemed the data from the entire region unusable. We repeated data collection in Multan with different interviewers employed under legal contracts that made them liable in the event of fraud or dishonesty. They were paid on delivery and successful verification of questionnaires, rather than on a monthly basis, removing the incentive of monetary gain by deliberately prolonging the data collection phase.

The two-day field visit for authentication of data was made after delivery of 300 of the required 800 questionnaires. We randomly selected 10% (80) from different clusters in Multan City and its adjoining rural areas. We disclosed the addresses of the selected households to the interviewers on the day of our visit. Interviewers were obliged by their contracts to accompany the co-investigator to these households. All 80 households were located, and their participants verified; all recognized their interviewers.

This re-survey in Multan was completed in 3 months. The fabricated data were withdrawn from the database and replaced with the new data. Table 1 compares the two datasets. The demographic data show a reversed male:female ratio and an unfeasible bimodal age distribution in the fraudulent dataset, with a migraine prevalence of 51.4%.

## Discussion

A recent review noted that scientific misconduct is on the rise [15]. Whether or not this is true (rather than increasing awareness of it – or greater willingness to recognize it), fabrication or modification of research data is clearly common [2] and can have far-reaching consequences. It is obvious that decisions based on falsified data regarding treatments, health-care priorities, health policy and health-resource allocation may be seriously misguided [3,8,9]. Future research unknowingly built upon fabricated data may be disastrously misled [10].

The usual motivation for falsifying research is monetary gain, either directly or, in academic circles, through career advancement [16]. In this case, simple laziness was an alternative explanation, but the truth was probably more complicated. In epidemiological research, committed investigators may plan and organize every step of a survey but data collection often depends on hired interviewers with no personal interest in the research. Not least because data collection is a time-consuming and commonly tedious process, vulnerability to fraud is high. It seems important to recognise this. Although a certain amount of trust is necessary for the implementation of a study, it is unfortunately but clearly necessary to implement quality checks [17]. The quality-assurance methods utilized here were pioneered in an LTB-sponsored study in India [18].

It is salutary to note that preventative measures alone were not sufficient here; detective measures were needed also. In this instance, the fraud was unsophisticated, and therefore readily detected – once it had been suspected. Successful data fabrication requires some understanding of what the data should look like, which the miscreant interviewers lacked. They were not, it seems also, practised fraudsters: they did not apparently employ the common technique (in fraud) of properly recording data from an initial relatively small sample and then reproducing these data repeatedly with minor changes – which produces a large dataset with a degree of verisimilitude (unless, by chance, the initial sample happened to be atypical). Nevertheless, without quality assurance, the Multan data might simply have been pooled with those from the other locations, and the discrepancies, though still misleadingly influential upon the survey as a whole, would not then have been obvious.

Quality assurance measures add to study costs, and national surveys are not done cheaply: human resource and travel costs are high. But the greater cost to us – both financially and in lost time – was in having to discard data from over 800 participants and repair the survey by repeating a large part of it [17].

We learnt some lessons. We would have done better at the outset to introduce legally-binding contracts rather than informal understandings, although this might not be true, or feasible, in all cultures. Interviewers should have been paid on successful delivery and after initial analysis of data, rather than on a monthly basis. Field visits probably would better have been conducted earlier during the data collection phase, although, since the problems arose with rural data collection, and most interviewers completed urban data collection first, this might have been falsely reassuring.

## Conclusion

Fraud in community-based surveys is seldom reported, but it occurs and it should not be assumed to do so less frequently than in other fields of research. This incident and its aftermath are reported to highlight the need for anticipation, prevention, detection and, when it is discovered, correction of fraud in future community-based interviewer-dependent surveys.

## Competing interests

TJS is a director and trustee of *Lifting The Burden*. He and the other authors report no other conflicts of interest.

## Authors’ contributions

BA and AA conceived the study. They and all other authors participated in the study and its analysis, were involved in drafting the manuscript and read and approved the final version.
